# Probiotics and periodontal health

**Published:** 2011-11-24

**Authors:** G Gupta

**Affiliations:** Department of Periodontics, Institute of Dental Studies and Technologies, Modinagar, UP India

**Keywords:** Probiotics, Synbiotics, Periodontitis, Bacterial replacement therapy, Halitosis

## Abstract

Periodontitis is one of the most common chronic inflammatory diseases. The etiology is clearly bacterial and a number of putative bacterial pathogens have been associated with the disease, including Aggregatibacter actinomycetemcomitans, Tannerella forsythus and Porphyromonas gingivalis. Comparatively, little attention has been paid to the identification of health-associated and potentially beneficial bacterial species that may reside in the gingival sulcus. Probiotic technology represents a breakthrough approach to maintaining oral health by using natural beneficial bacteria, commonly found in healthy mouths, to provide a natural defense against those bacteria which are thought to be harmful to teeth and gums. This article endeavors to introduce the concepts of probiotics in periodontics.

## Introduction

Periodontitis is a multifactorial disease that encompasses the hard and soft tissue, microbial colonization (with or without invasion), inflammatory responses and adaptive immune responses. The complexity of the local tissue components, including bacteria and/or their products and virtually all aspects of host response mechanisms, has complicated our ability to elucidate the critical protective functions in the tissues and has continually provided evidence for the potential of host destructive factors as the ultimate causative parameters in the disease. [**1**]Conventional treatment modalities of periodontal disease include non-surgical and surgical management, which emphasizes mainly on mechanical debridement, often accompanied by antibiotics. These treatment modalities are aimed at eliminating the entire microflora irrespective of their pathogenicity. Due to the emergence of antibiotic resistance and frequent recolonization of treated sites with pathogenic bacteria, there was need for a new treatment paradigm to be introduced to periodontal disease. The need was fulfilled by introduction of Probiotics and bacterial replacement therapy in the field of periodontics.

## Probiotics and prebiotics

Probiotics are defined as living microorganisms, principally bacteria, that are safe for human consumption and, when ingested in sufficient quantities, have beneficial effects on human health, beyond basic nutrition. This definition has been approved by the United Nations Food and Agriculture Organization (FAO) and the World Health Organization (WHO). [**2**]

Prebiotics (i.e. inulin-type fructans, maltodextrin, fructooligosaccharides and galactooligosaccharides) have been defined as non-digestible oligosaccharides that affect the proliferation of resident commensal bacteria that may then exert probiotic effects. [**3**] More recently, the definition has been refined to include selectively fermented ingredients that allow specific changes in the composition and/or activity of the resident microflora that confer benefits upon host well-being and health. [**4**]

Prebiotics and probiotics often work in synergy and, when combined in the same product, are known as synbiotics. It appears that synbiotics increase survival of probiotic bacteria, stimulating their growth in the intestinal tract and improving the balance of health-promoting bacteria. [**5**]

### History

The dietary use of living microorganisms has a long history. Mention of cultured dairy products is found in the bible and the sacred books of Hinduism. Sacred milks and cultured dairy products such as *kefir, koumiss, liben and dahi*, were often used therapeutically before the existence of microorganisms was recognized.[**6**]

Probiotics have been used for decades in fermented products, but potential use of probiotics as a nutritional medical therapy has not been formally acknowledged. [**7**] Metchnikoff was the first to state that probiotics could provide a health benefit, and proposed that Bulgarian people had a longer longevity due to fermented milk containing viable bacteria. "Probiotic" term, as opposed to "antibiotic", was initially proposed by Lilley and Stillwell in 1965. First probiotic species to be introduced in research was Lactobacillus acidophilus by Hull et al. in 1984; followed by Bifidobacterium bifidum by Holcombh et al. in 1991.

In 1994, the World Health Organization deemed probiotics to be the next-most important immune defense system when commonly prescribed antibiotics are rendered useless by antibiotic resistance. These incidences paved way for a new concept of probiotics in medicine and dentistry. [**8-10**]

### Mechanisms of action

#### Probiotics

The mechanism of action vary according to the specific strain or combinations of strains used, the presence of prebiotics and the condition that is being treated, as well as the stage of the disease process in which the probiotic is administered. [**11**] There are common themes emerging in studies of the modes of action of probiotics and numerous mechanisms have been proposed [**11,12**] including: • Inhibition of pathogen adhesion, colonization and biofilm formation • Induction of expression of cytoprotective proteins on host cell surfaces • Inhibition of collagenases and reduction of inflammation associated molecules • Stimulation and modulation of the host immune system, e.g. by reducing production of pro-inflammatory cytokines through actions on NFκB pathways, increasing production of anti-inflammatory cytokines such as IL-10 • Modulation of cell proliferation and apoptosis eg. Prevention of cytokine induced apotosis • Killing or inhibition of growth of pathogens through production of bacteriocins or other products, such as acid or peroxide, which are antagonistic towards pathogenic bacteria • Probiotics can also modify the surrounding environment by modulating the pH and/or the oxidation–reduction potential, which may compromise the ability of pathogens to become established

#### Prebiotics

The major mechanism of action of prebiotics is assumed to be indirect, i.e. facilitating the proliferation of beneficial components of the resident microflora. Some prebiotics also exert direct effects on the host; independent of their effects on resident bacterial populations. [**13**] These include stimulation of expression of IL-10 and interferon γ, enhancement of IgA secretion, modulation of inflammatory responses to pathogens and stabilization of the gut mucosal barrier. Additionally, prebiotics with enhanced function have been designed. These oligosaccharide derivatives contain sugars that are specific epithelial cell receptors to which pathogens adhere and they, therefore, provide „decoy” adhesion sites and cause pathogens to adhere to luminal contents rather than to epithelial cells. [**14**]

### Oral microbiota in health and disease

More than 700 species have been detected in the human mouth and the resident microbiota of an individual may comprise 30 to >100 species.15 A wide variety of sites in the mouth are heavily colonized. Supragingival and subgingival plaque form through sequential and specific adhesive interactions that result in a complex climax community. [**16**] The tongue is heavily colonized and micro-organisms on the dorsum of the tongue are reservoirs for supragingival and subgingival plaque and salivary microbial populations. [**17**] Many oral bacteria, especially streptococci, also survive within buccal epithelial cells. [**18**]

### Functions of the resident microbiota

Resident microbiota does not play merely a passive role, but actively contributes to the maintenance of health. The large, diverse resident microbial communities that colonize mucosal sites co-exist with a host, producing harmful effects only if the host becomes immunocompromised, if the resident microbial populations are suppressed or if micro-organisms reach sites to which they do not normally have access (i.e. through trauma). Resident microbial populations contribute to host protection through development of the immune system, [**19**] the maintenance of healthy oral tissue by influencing expression of mediators such as intracellular adhesion molecule 1 (ICAM-1), E-selectin and IL-8, [**20**] modulating immune responses and enhancing cellular homeostatic mechanisms. [**21**]

### Defining the resident microbiota

Resident oral microbial populations are site-specific as well as highly diverse. Kilian et al. [**22**] list the following species as „true” oral commensal microorganisms: Streptococcus mitis, Streptococcus oralis, Actinomyces naeslundii, Fusobacterium nucleatum, Haemophilus parainfluenzae, Eikenella corrodens and some species of Prevotella.

### Microbial populations associated with oral disease

The most common oral diseases are caries and periodontitis, which result from a shift in the balance of the resident microbiota at a site. In caries, there are increases in acidogenic and acid-tolerating species such as mutans streptococci and lactobacilli, although other bacteria with similar properties can also be found and bifidobacteria, non-mutans streptococci, Actinomyces spp., Propionibacterium spp., Veillonella spp. and Atopobium spp. have also been implicated as significant in the etiology of this disease. [**23**]

In periodontal diseases, there is an increase in plaque mass and a shift towards obligatory anaerobic and proteolytic bacteria, many of which are Gram negative and currently unculturable. The host damage that occurs during periodontal disease arises through the combined activities of subgingival biofilms and the host responses to these diverse bacterial populations. [**16**]

Candida albicans and other Candida species are present in low levels in oral microbial communities and can cause oral candidiasis and denture- associated stomatitis. [**24**] Halitosis is most often the result of production of malodorous metabolic endproducts (especially volatile sulphur compounds) by oral bacteria, in particular Gram negative anaerobes.[**25**]

### Probiotics of interest

The most common probiotic strains belong to the genera *Lactobacillus* and *Bifidobacterium*. *Lactobacillus* species from which probiotic strains have been isolated include *L. acidophilus, L. johnsonii, L. casei, L. rhamnosus, L. gasseri, and L. reuteri.* Similarly, the *bifidobacterium strains include B. bifidum, B. longum, and B. infantis. Lactobacilli* can produce different antimicrobial components including organic acids, hydrogen peroxide, low-molecular weight antimicrobial substances, bacteriocins and adhesion inhibitors and thus have gained prominence as probiotics. *Streptococcus Oralis* and *Streptococcus Uberis* have been shown to inhibit the growth of pathogens both in the laboratory and animal models. [**26**]

### Probiotics in prevention of periodontal diseases

Periodontal disease is classified into 2 types: gingivitis and periodontitis. Gingivitis is characterized by inflammation limited to the gingiva, whereas periodontitis is a progressive, destructive disease that affects all supporting tissues of the teeth, including the alveolar bone. [**27**] The main pathogenic agents associated with periodontitis are *P. gingivalis, Treponema denticola, Tannerella forsythus* and *Aggregatibacter actinomycetemcomitans*. [**27**] These bacteria have a variety of virulent characteristics allowing them to colonize the subgingival sites, escape the host”s defense system and cause tissue damage. [**27**] The persistence of the host”s immune response also constitutes a determining factor in progression of the disease. [**27**]

There are fewer experimental studies exploring probiotic use in periodontal diseases, partly reflecting a poorer understanding of the precise etiology of the disease and of the conditions that promote health. Grudianov et al [**28**] studied the effect of probiotic tablets on gingivitis and different grades of periodontitis and observed that probiotic treatment resulted in better microbiota normalization than control group. In one recent study, the prevalence of lactobacilli, particularly *L. gasseri* and *L. fermentum*, in the oral cavity was greater among healthy participants than among patients with chronic periodontitis. [**29**] Various studies have reported the capacity of lactobacilli to inhibit the growth of periodontopathogens, including *P. gingivalis, Prevotella intermedia* and *A. actinomycetemcomitans*. [**29**] Together, these observations suggest that lactobacilli residing in the oral cavity could play a role in the oral ecological balance.

In a study by Krasse et al, [**30**] patients with moderate to severe gingivitis who were given either one of two L. reuteri formulations had reduced plaque and gingivitis scores compared to a placebo group. Although the exact mechanisms of action of *L. reuteri* remain to be elucidated, previous studies have suggested at least 3 plausible possibilities: first, *L. reuteri* is known for its secretion of 2 bacteriocins, reuterin and reutericyclin, that inhibit the growth of a wide variety of pathogens; [**31**] second, *L. reuteri* has a strong capacity to adhere to host tissues, thereby competing with pathogenic bacteria; [**32**] and third, the recognized anti-inflammatory effects of *L. reuteri* on the intestinal mucosa, leading to inhibition of secretion of proinflammatory cytokines, could be the foundation for a direct or indirect beneficial effect of this bacterium on people with periodontal disease. Vivekanda et al [**33**] also confirmed the plaque inhibitory, anti-inflammatory and antimicrobial effects of *L. reuteri* Prodentis.

Riccia and colleagues [**34**] recently studied the anti-inflammatory effects of *Lactobacillus brevis* in a group of patients with chronic periodontitis. A significant reduction in salivary levels of prostaglandin E2 (PGE2) and matrix metalloproteinases (MMPs) was observed. The authors suggested that the beneficial anti-inflammatory effects of *L. brevis* could be attributed to its capacity to prevent the production of nitric oxide and, consequently, the release of PGE2 and the activation of MMPs induced by the nitric oxide. [**34**] However, *L. brevis* may also be antagonistic, leading to a reduction in the quantity of plaque and therefore an improvement in the gingival index

Shimauchi et al [**35**] reported that the regular (three times daily for eight weeks) intake of tablets containing Lactobacillus salivarius resulted in benefits in terms of pocket probing depth and plaque index in individuals at high risk of periodontal disease (smokers) compared to a placebo control group. [**35**] Other studies have aimed to identify organisms that have the potential for probiotic action that may protect against periodontal diseases. Some oral strains of lactobacilli and streptococci [**36**] and bifidobacteria [**37**] have been reported to have in vitro inhibitory activity against periodontal pathogens, while others are more active against mutans streptococci. [**36**] Koll-Klais et al [**38**] observed that *Lactobacillus gasseri* strains isolated from periodontally healthy subjects were more efficient at inhibiting the growth of *A. actinomycetemcomitans* than strains from periodontally diseased subjects. *L. gasseri* also inhibited the growth of *P. gingivalis* and *P. intermedia*; this correlated with an inverse relationship between carriage of homofermentative lactobacilli and subgingival colonization by *A. actinomycetemcomitans, P. gingivalis* and *P. intermedia*. Ishikawa et al. [**39**] observed in vitro inhibition of *P. gingivalis*, *P. intermedia* and *Prevotella nigrescens* by *L. salivarius*. Daily ingestion of *L. salivarius*-containing tablets resulted in reduced salivary counts of these black pigmented anaerobes. The mechanisms of inhibition of periodontal pathogens have not been fully clarified. The inhibitory activity displayed by homofermentative lactobacilli against periodontal pathogens was principally related to their production of acid, not to H2O2 or bacteriocin production. [**38**] Hojo et al [**37**] suggested that bifidobacteria inhibit some black pigmented anaerobes by competing for an essential growth factor, vitamin K, although there was no significant relationship between higher bifidobacterial counts and lower black-pigmented anaerobe counts. Recently, a bacteriocin purified from *Lactobacillus casei* killed *P. gingivalis* but its use was proposed as a novel chemotherapeutic agent rather than as strain development for probiotic applications.[**40**] During the fermentation process in milk, *Lactobacillus helveticus* produces short peptides that act on osteoblasts and increase their activity in bone formation. [**41**] These bioactive peptides could thereby contribute to reducing the bone resorption associated with periodontitis.

Recently Shimazaki and colleagues [**42**] used epidemiological data to assess the relationship between periodontal health and the consumption of dairy products such as cheese, milk and yoghurt. The authors found that individuals, particularly nonsmokers, who regularly consumed yoghurt or beverages containing lactic acid exhibited lower probing depths and less loss of clinical attachment than individuals who consumed few of these dairy products. A similar effect was not observed with milk or cheese. By controlling the growth of the pathogens responsible for periodontitis, the lactic acid bacteria present in yoghurt would be in part responsible for the beneficial effects observed.

Sunstar (Etoy, Switzerland) recently began marketing the first probiotic specifically formulated to fight periodontal disease. Gum Perio Balance contains a patented combination of 2 strains of *L. reuteri* specially selected for their synergetic properties in fighting cariogenic bacteria and periodontopathogens. Each dose of lozenge contains at least 2 × 108 living cells of *L. reuteri* Prodentis. Users are advised to use a lozenge every day, either after a meal or in the evening after brushing their teeth, to allow the probiotics to spread throughout the oral cavity and attach to the various dental surfaces.

### Guided periodontal pocket recolonization (Bacterial replacement therapy) in Periodontics

“Replacement therapy” is also known as “probiotic therapy”. The concept of bacterial replacement therapy in periodontics was first introduced by Teughels et al in 2007. They reported that the subgingival application of a bacterial mixture including *Streptococcus sanguis, S. salivarius*, and *Streptococcus mitis* after scaling and root planing significantly suppressed the re-colonization of *Porphyromonas gulae* (canine *P. gingivalis*) and *P. intermedia* in a beagle dog model. [**43**] Nackaerts et al [**44**] observed that the subgingival application of beneficial oral bacteria (i.e. *Streptococcus sanguinis, Streptococcus salivarius and S. mitis*) delays recolonization by periodontal pathogens, reduce inflammation, and improve bone density and bone levels in a beagle dog model. This guided pocket recolonization approach may provide a valuable addition or alternative to the armamentarium of treatment options for periodontitis.

### Probiotics in prevention of halitosis

Halitosis has many causes (including consumption of particular foods, metabolic disorders, respiratory tract infections), but in most cases it is associated with an imbalance of the commensal microflora of the oral cavity. [**45**] More specifically, halitosis results from the action of anaerobic bacteria that degrade salivary and food proteins to generate amino acids, which are in turn transformed into volatile sulphur compounds, including hydrogen sulphide and methyl mercaptan and dimethyl sulphide. [**45**]

There have also been clinical and laboratory studies of probiotics in their potential for preventing halitosis. Peroxide production by strains of *Weissella cibaria* (commonly present in fermented foods) isolated from the mouths of healthy children, inhibited production of volatile sulphur compounds that contribute to oral malodour by *F. nucleatum* in vitro and in exhalations following mouth-rinsing by adult volunteers with a suspension of *W. cibaria*. [**46**] The success of *W. cibaria* in reducing malodour may have also been because it coaggregated efficiently with *F. nucleatum* [**46**] and therefore competed with other late/secondary colonizers for adhesion sites. Thus, *W. cibaria* may have probiotic activities with potential for prevention of periodontal disease. Volatile sulphur compounds, such as H2S and mercaptoethanol, are produced by a range of periodontal anaerobes. [**47**] The inhibition of these micro-organisms by peroxide from *W. cibaria* may help reduce subgingival plaque pathogenicity while its competition for coaggregation sites may reduce the reservoir of micro-organisms available for transmission into plaque.

One recent study [**48**] showed that certain bacterial species, including *Atopobium parvulum, Eubacterium sulci* and *Solobacterium moorei*, predominate on the dorsal surface of the tongue among people with halitosis. Conversely, another species, *Streptococcus salivarius*, was detected most frequently among people without halitosis and is therefore considered a commensal probiotic of the oral cavity. *S. salivarius* is known to produce bacteriocins, which could contribute to reducing the number of bacteria that produce volatile sulphur compounds. [**49**]

*S. salivarius* K12 produces salivaricin, a lantibiotic with inhibitory activity towards most Streptococcus pyogenes. [**50**] This strain has been commercially promoted as a probiotic that is reported to be protective against throat infections and oral malodour. [**50**] The importance of strain selection for probiotic use is illustrated by the fact that some *S. salivarius* strains differ from K12 in some important activities; one strain increased production of malodorous products by facilitating *P. gingivalis* metabolism of salivary mucins [**51**] and another up-regulated IL-8 secretion by oral epithelial cells [**52**] in contrast to the down-regulation observed in response to K12.

### Residence time of probiotics in oral cavity

Residence time of probiotics in oral cavity after treatment withdrawal was studied by Çaglar et al. [**53**] A reduced *S. mutans* level was shown after a two-week use of a *L. reuteri-enriched* yoghurt; effects were observed during use and for a few days after discontinuation. A loss of *L. reuteri* colonization was observed by Wolf et al. [**54**] two months after having discontinued probiotic use. *L. rhamnosus* GG administration and oral cavity colonization was studied by Yli-Knuuttila et al [**55**]; the authors concluded that permanent colonization in oral cavity was unlikely (although possible in some cases) and suggested the probiotic to be used on a regular basis. Binding strength of 17 Lactobacillus strains and 7 bifidobacteria strains to saliva and oral mucous membrane was variable in different strains, according to a study by Haukioja et al. [**56**] Such a strength variation caused an increased residence time of probiotic in oral cavity. Latency time of probiotic *S. salivarius* K12, 4 tablets/day for 3 days, was assessed in several oral cavity areas in a 35-day follow-up, by Horz et al. [**57**] Probiotic could be found on oral mucous membrane, tongue and in stimulated saliva for more than 3 weeks, with a gradually reduced *S. salivarius* K12 level being detected beginning 8 days after treatment withdrawal.

### Potential risks of probiotic therapy

Different strains of a species may not all possess characteristics that enable them to be probiotics and rigorous strain selection for the disease concerned is complex but essential. [**58**] Some probiotic strains have been in use for many years and have excellent safety records. [**59**] Most probiotic bacteria are weakly proteolytic and, for example, *Lactobacillus bulgaricus*, was shown to be incapable of degrading some host tissue components. [**60**] However, there have been some cases of bacteraemia and fungaemia associated with probiotic use, although these have been in subjects who are immunocompromised, [**61**] or who suffer from chronic disease [**59**] or short gut syndrome. [**62**] Other predisposing factors include prior prolonged hospitalization and prior surgical intervention. [**61**] An individual who had been taking *L. rhamnosus* in a probiotic preparation developed Lactobacillus endocarditis following dental treatment. [**63**] In Finland, however, there has not been an increase in bacteremia associated with probiotic lactobacilli following the increase in the use of these products since 1990. [**64**] The species that most commonly exhibit probiotic benefits are lactobacilli and other lactic acid bacteria, and the production of acid is often thought to be an important component of their protection against pathogenic colonization. However, *Lactobacillus spp.* and acid production by acidogenic plaque populations play a significant part in the development of caries, and a probiotic strain of *L. salivarius* has been shown to be cariogenic in a rat model. [**65**] A number of probiotic lactobacilli and bifidobacteria produce acid from fermentation of dietary sugars in vitro. [**66**] There are conflicting data on the salivary lactobacilli levels following probiotic usage. Some studies have reported no effects, [**67**] others have found trends for an increase, [**68**] while others have detected statistically significant rises in counts of salivary lactobacilli. [**69**] There is a converse risk in that the control or prevention of caries may indirectly affect periodontal pathogens. It has been known for many years that streptococci, through production of hydrogen peroxide, inhibit the growth of putative periodontal pathogens, leading to early proposals that interactions between groups of micro-organisms within plaque can influence the development of disease or actively contribute to the maintenance of health, [**70**] and lactobacilli and bifidobacteria also inhibit the growth of a range of periodontal pathogens. [**70**] It is clear that careful selection of the strain to be ingested for a particular disease is essential and the mode and timing of administration can be crucial, as well as the age and health of the individual taking the probiotic. There is a sufficient knowledge base for major and minor risk factors to have been proposed for administration of probiotics to prevent intestinal conditions, [**59**] but this knowledge base for oral applications is clearly more distant. One of the biggest problems to overcome may be that the probiotic activities and micro-organisms that protect against oral disease could increase the risk of development of dental caries. Therefore, a prebiotic-type approach to enhance endogenous beneficial commensals may be more attractive. It will also be a challenge to ensure that modes of delivery are developed that provide sufficient retention and exposure times in the mouth that will allow probiotics to colonies plaque or prebiotics to enter into plaque or mucosal biofilms and influence microbial metabolism within them.

## Conclusion

Probiotics are emerging as a fascinating field in oral medicine. This concept prompts a new horizon on the relationship between diet and oral health. The use of probiotics for use in oral care applications is gaining momentum. There is increasing evidence that the use of existing probiotic strains can deliver oral health benefits. Further work will be needed to fully optimize and quantify the extent of this benefit. In parallel, the potential of prebiotics to maintain and enhance the benefits provided by the resident oral microbiota will be investigated. However, whether considering probiotics or prebiotics, it will be essential to develop an understanding of the broad ecological changes induced in the mouth by their ingestion and the long-term consequences of their use on oral health and disease. Further studies to understand the ability of probiotic bacteria to survive, grow, and have a therapeutic effect when used for treatment or when added to foods, to fix the doses and schedules of administration of probiotics. Hence, systematic studies and randomized controlled trials are needed to find out the best probiotic strains and means of their administration in different oral health conditions. Finally, possibilities to genetically modify or engineer potential probiotic strains may offer all new visions. Better scientific understanding and extended research of these tiny forms of life and their effect on humans in the treatment of periodontal diseases might further broaden the field of potential applications.
